# Editorial: Cholesteatoma surgery: treatment outcome and follow up

**DOI:** 10.3389/fsurg.2026.1834766

**Published:** 2026-04-17

**Authors:** Hans Thomeer, Paul Merkus, Nicolas Verhaert, Marc Lammers

**Affiliations:** 1 Department of Otorhinolaryngology, Utrecht University, Utrecht, Netherlands; 2 Universitair Medisch Centrum Utrecht, Utrecht, Netherlands; 3 UMC Utrecht Brain Center Rudolf Magnus, Utrecht, Netherlands; 4 Department of Otorhinolaryngology, Katholieke Universiteit Leuven, Leuven, Belgium; 5 Department of Otorhinolaryngology, Amsterdam UMC Locatie AMC, Amsterdam, Netherlands; 6 Amsterdam UMC Locatie VUmc, Amsterdam, Netherlands; 7 Department of Otorhinolaryngology, UZ Leuven, Leuven, Belgium; 8 Department of Otorhinolaryngology, Universitair Ziekenhuis Antwerpen, Edegem, Belgium; 9 Universiteit Antwerpen, Antwerp, Belgium

**Keywords:** cholesteatoma, hearing loss, iatrogenic, recurrence, surgery

This Research Topic focuses on the pathophysiology of chronic otitis media cholesteatoma and its treatment. The origin and etiology of the pathology is still under debate and not the focus of this Research Topic. Four articles can be viewed and studied by the reader to further understand the impact on hearing and speech perception due to the disease sequalae. Surgery is the most common form of treatment, and three research groups (Zhao and Ye, Karkas et al., and Erfurt et al.) describe suboptimal hearing recovery to within normal ranges (i.e., air conduction levels below 20dB PTA) after long-term postoperative follow up. However, how to improve hearing following ossicular chain reconstruction remains a subject of ongoing scientific investigation. Although these three articles provide the reader with valuable additional hearing outcome data and provide new insights regarding the value of the mastoid obliteration technique in reducing disease recurrence, the next step in cholesteatoma treatment is still to be established.

In that respect, the article by Mahmoud et al. might provide a glimpse into the future with their discussion of an innovative drug delivery support and the potential of porphysomes; applying these new photothermal agents during cholesteatoma surgery seems to aid in the total eradication of the disease. New and prospective clinical trials will further elucidate the added value of these techniques.

